# The Women’S Perceptions About Unwanted Pregnancy: A Qualitative Study in Iran

**DOI:** 10.5539/gjhs.v8n5p189

**Published:** 2015-09-28

**Authors:** Marzieh Akbarzadeh, Zahra Yazdanpanahi, Ladan Zarshenas, Farkhondeh Sharif

**Affiliations:** 1 Maternal–Fetal Research Center, Department of Midwifery, School of Nursing and Midwifery, Shiraz University of Medical Sciences, Shiraz, Iran; 2 Department of Midwifery, School of Nursing and Midwifery, Shiraz University of Medical Sciences, Shiraz, Iran; 3 Department of Mental Health and Psychiatric Nursing, School of Nursing and Midwifery, Community Based Psychiatric Care Research Center, Member of Quality Improvement in Clinical Education Research Center, Shiraz University of Medical Sciences, Shiraz, Iran; 4 Department of Mental Health and Psychiatric Nursing, Community Based Psychiatric Care Research Center, School of Nursing and Midwifery, Shiraz University of Medical Sciences, Shiraz, Iran

**Keywords:** experience, qualitative research, unwanted pregnancy, women

## Abstract

**Background:**

Unwanted pregnancy has affected different aspects of our life. Researchers point out if a child’s birth is unwanted, an increase in maternal issues can be observed. The aim of this study was to describe the women’s viewpoints regarding unwanted pregnancy.

**Method:**

This qualitative study using content analysis was employed in Shiraz University in 2013-2014. 20 women with unwanted pregnancy were chosen using purposeful sampling. Data were gathered through semi-structured interviews and trustworthiness of them was evaluated.

**Results:**

Findings of this study, according to the participants’ experience, revealed maternal emotions like embarrassment for getting pregnant, mother’s own negative affection, concerns about missing family and other children, and terminating the pregnancy through illegal abortion, the husband’s disagreement about the termination, blaming themselves for thinking about abortion or illegal abortion, and concern about their guilt feeling. Another finding was family problems like husband’s behavior and his negative outlook towards his child and fetus. The next category belonged to anxiety about the future of their child, including economic, social and relational problems, and suppression of the children’s logical expectations. The last finding was the lack of maternal emotional support.

**Conclusions:**

Unwanted pregnancy’s effects on the mothers and infants’ health are considerable. A closer observation by family and health care providers for unwanted pregnancies and its results is recommended; therefore, they should be taken care of as high risk pregnancies, requiring family support.

## 1. Introduction

Unwanted pregnancy is a universal issue which affects women, their families and the society (Klima, 1998). It occurs in every social and economic status. Almost 50% of pregnancies in the United States are unwanted and approximately 48% of women between 15 to 44 years of age have experienced unwanted pregnancy at least for once (Moos, 2003). 50 millions of unwanted pregnancies would lead to intentional abortion each year, and it is estimated that 42 million abortions happen annually, among which 22 million are safe and 20 million are unsafe. In illegal abortion, 70000 fatalities among mothers and over 5 million temporary or permanent disabilities have been reported (Shah & Ahman, 2009). In almost, 9 million cases observed in developing countries, more than 200 fatalities of women are seen each day. Mothers who are eager to get pregnant have a better psychological preparation for facing socio-physical issues and difficulties of delivery. Also, they check themselves up for health care, and will not hesitate in doing the health care providers’ instructions to keep themselves and their fetus healthy. Therefore, such pregnancies will have desirable results (Myhrman, Rantakallio, Isohanni, Jones, & Partanen, 1996). On the contrary, women with unwanted pregnancies adapt fewer healthy behaviors. Behaviors such as smoking and postponing pregnancy care, which are risk factors for low birth weight and age, are more common among these women (Campo, Askelson, Spies, & Losch, 2010). Women with unplanned pregnancies demonstrated a significantly more disturbed mood, both in pregnancy and in the first year after the birth (Olukoya, Kaya, Ferguson, & AbouZahr, 2001). Studies indicate that unwanted pregnancy is accompanied by an increase in unhealthy abortion, early delivery and low birth weight (Saeedi & Ahmadian, 2013; P. S. Shah et al., 2011; Tehrani, Allameh, & Mehrabi, 2014). Women with unwanted pregnancy face more violence by their husbands as opposed to women with planned pregnancy. Furthermore, the most significant reason of suicide and depression among women with an unwanted pregnancy is the limitation in selection in this field and an increase in stillbirth and infants’ fatality which is two times as much as that in planned pregnancies (Wewerinke, Honig, Heres, & Wennink, 2006). Unwanted pregnancies are more observed in young women and young adults(Ikamari, Izugbara, & Ochako, 2013).

Such women have adverse pregnancy outcomes and suffer more for their infants and physical and psychological issues and experiences during their childhood (Kroelinger & Oths, 2000). Regions in which abortion is illegal are faced with an increase in illegal abortions and it is a major urban phenomenon in many advanced and developing countries today. In recent studies, 67% of women with imperfect abortion had had an unwanted pregnancy (Rasch, Yambesi, & Kipingili, 2005). In addition to physical aspects mentioned above, most mothers face psychological crisis. In other words, the basis of their physical disorders in unwanted pregnancy is their attitude, emotions and anxiety. Unwanted pregnancy leads to a negative outlook towards pregnancy (Rasch et al., 2005) and abusing the process of parental role in evolution, which is formed during pregnancy (Waldenström, Hildingsson, Rubertsson, & Rådestad, 2004). It is necessary to study thoughts, beliefs and ideas of women and other people involved in unwanted pregnancy so as to sensitize people to its consequences which is taken into account in few studies. Therefore, this study aimed to assess and understand the women’s perspectives about unwanted pregnancy. Analyzing mothers’ opinions might lead to knowledge on how to prevent the consequences of such pregnancies and at least manage a healthy pregnancy. The results of this study could be a basis for planning for health care systems in order to reduce the side effects and consequences that affect physical, psychological and emotional health. Hence, this study could be practicable in a midwifery-clinical environment. By obtaining such information, midwives and obstetricians could help mothers with unwanted pregnancies more effectively.

### 1.1 Abortion and Unwanted Pregnancy in Iran/Shiraz

In Iran, only abortion in certain cases such as saving the life of the mother or malformation of the embryo is allowed (Bazmi, Behnoush, Kiani, & Bazmi, 2008). Abortion imposes high direct and indirect costs on the healthcare system and the family in any country. An abortion in Iran is estimated to be about $200. Official statistics about abortion in Iran does not exist (Motaghi, Keramat, Shariati, & Yunesian, 2013). However, it has been pointed out in the reports of various studies. In a meta-analysis study on the prevalence of unwanted pregnancy, miscarriage was reported 27.94% per 100 pregnancies (Motaghi, Poorolajal, et al., 2013). In another study, it was reported 31% (Moosazadeh, Nekoei-Moghadam, Emrani, & Amiresmaili, 2013). In Shiraz, from 596 pregnant women in 16 active clinics in 2010, about 9.24% unwanted pregnancy were reported (Akbarzadeh & Alizadeh, 2010).

## 2. Method

### 2.1 Study Design and Population

The present research is a qualitative study using content analysis, which is a research method with the purpose of providing knowledge and understanding about the phenomena under the study (Hsieh & Shannon, 2005), new insights, a representation of facts, and practical guide to action (Elo & Kyngäs, 2008). It provides a systematic and objective means in order to describe the phenomena and is concerned with meanings, intentions, consequences and context (Cavanagh, 1997). In this study, data were collected about 20 women with unwanted pregnancies who referred to Hafez educational-clinical center in Shiraz University in 2013- 2014. Purposeful sampling method using semi-structured interviews were performed, lasting about 30 to 45 minutes. Interviews began with a general question such as “What is your experience about unwanted pregnancy?” and continued with questions such as “How do you feel about your present pregnancy?”, “How will you feel about your future (after delivery and later)?”

The inclusion criteria were mothers who had experienced unwanted pregnancy, a minimum time of 3 or 4 months passed from their pregnancy, being in their second pregnancy or more, and having no commonly known disease (diabetes, heart-kidney disorders). Those whose husband were reluctant about pregnancy and those with their first pregnancy were excluded. Data were analyzed using Max qda 2007.

### 2.2 Ethical Considerations

The ethics committee in Shiraz University of Medical Sciences approved the study proposal. The aims of the study and the voluntary nature of the participants, anonymity, confidentiality and also right to withdraw from the study were explained and then a written informed consent was obtained.

### 2.3 Data Analysis

The data were analyzed using content analysis. The researcher recorded the interviews varying in time from 30-45 min. After that, each recorded interview was transcribed and read several times. The interviews were divided into condensed meaning units. Each condensed unit was labeled with a code. Similar codes and those having the same meanings were grouped together and formed subcategories. Then, the subcategories were combined to create categories.

### 2.4 Trustworthiness

The credibility of the data was confirmed by member check with participants to assess the researchers’ understanding of their ideas and peer check by researchers. For dependability in the depth, methodological description was used and for transferability, thick description of the phenomenon was provided.

## 3. Results

Based on the obtained results of the research, 20 women, all of whom had experienced unwanted pregnancy, participated in the study. The number of pregnancies was between two to eighth time pregnancy with the maximum frequency of the third time pregnancy (50%) and a minimum of 8th times (5%). This research indicated that reactions and experiences of the pregnant mothers towards unwanted pregnancy had several dimensions including physical problems, psychological reactions, family problems and socio-economic and nurturing problems. As to the participants’ ideas, unwanted pregnancy leads to physical problems such as pregnancy with bleeding, spotting, severe backache, dizziness and visual disturbance, lack of appetite for eating, picking up heavy things such as carpet, gas capsule for abortion which were along with severe backache. Also, it was found that it leads to psychological reactions, including feelings of psychological sorrow, stress and psycho-emotional issues of the husband and women. One mother [1] said:

“I was shy that I got pregnant; I felt embarrassed to be pregnant”. Another one [3] about negative reaction towards pregnancy stated:

“I hate myself because I was ignorant. Even I tried to commit suicide; lack of culture put me into this, and I feel inefficient” and the other one [11] said: “I have felt upset because of disregarding my other children and family member’s rights. I feel I have been cruel to my family and my other children, but I tried to find a person or a place to have an abortion and difficulty in providing medical and herbal medicine for my abortion. I had saffron, rosewater or other herbs so that they can help me to abort”. And “now I‘m worried about my baby because of the use of these herbs.” And the sixteenth mother said about having guilt feelings after trying for abortion

“I felt if I abort my child, I have murdered it. I was afraid that if I abort my baby, I have to pay for it; I had nightmares till 5- 6 months after having a shot and I wanted to see a psychologist”.

One mother [10] said about the tension and guilt feeling resulting from doubt and hesitation in abortion: “I was afraid of God to abort my baby”.

Being worried about the mother’s health was another finding; one mother [8] said “I have negative thoughts. What if something happens during surgery; I am over 40 years old and I am afraid that my baby and I would not have been healthy”.

Sleeping disorders were another category in this study. One mother [15] said:

“I had nightmares till 5-6 months of pregnancy; I woke up suddenly. I was stressed and I had low blood pressure and abdominal pain (nervous abdomen). I thought a lot and then I ended up crying. I was terrified about having a hard delivery”.

Some mothers had psychological anxiety and emotions towards the fetus and had a miserable feeling towards the existence of a growing fetus. One mother [18] said:

“During pregnancy, I hate my baby. Growing it up is a torture and I think after delivery I would even be more stressed than now”.

Being worried about her and her husband’s age must also be considered. One mother[10] said

“I feel sorry for my baby. When it is born, it will not be having young parents who are not patient with childhood naughtiness”.

Based on the participants’ ideas, family problems such as husband’s abusive behavior towards his pregnant wife was another reason for mothers’ tension. One participant [7] stated:

“My husband didn’t talk to me for one month after pregnancy. He blamed me for getting pregnant (husband’s conflict with his wife about why she hasn’t prevented it) and less family communications”.

Another family problem was anxiety of other children towards the fetus. One mother [13] said “My older children are very upset. They insist on aborting the baby. They say it is very offensive”.

Finally, socio-economic and nurturing problems were the possible reactions. Economic issues have been one of the other concerns which causes several problems based on participant’ ideas.

Inability to nurture children is one of the concerns. One participant [8] said:

“I am worried that I can’t nurture them well and I cannot be a good mother”.

And the other one [15] believed that:

“I am worried about my children’s future and I can’t support my children financially”. “I couldn’t register them in a good school; I am concerned about the costs”. One mother [17] said:

“If the number of children was low, the costs were allocated to the other children, having more shares”

According to the participants’ ideas, their communication with other children will be declined and expectation of them will be suppressed. One mother [9] said: “I feel I cannot fulfill my children’s expectations “and I am unfair toward them, I do not have time to satisfy their needs.” And the other one [9] said” When there are a lot of children, I will have less attention to them. I feel I am cruel to them”.

## 4. Discussion

The results of this study indicated that the women’s perceptions in unwanted pregnancy were physical problems, psychological reactions, family problems, socio-economic and nurturing problems. From the participants’ viewpoints, unwanted pregnancy affects the mother’s health in two ways of physical problems and psychological reactions. In our study, the participants experienced psychological reactions towards terminating the pregnancy and abortion. A great number of these couples thought of abortion, but most of them didn’t do anything about it. Some of them attempted it, but only a few were successful. Several factors such as socio-cultural beliefs, couple’s lifestyle, and husbands and health service providers had important roles in the process of decision making. Aborting a fetus is not just related to its physical side effects. The outstanding issue is its moral issues and hurting the human dignity and ego. Studies on women aged 40-45 years show that among the group who had unwanted pregnancy, 58% could achieve the abortion and 9% did not and 32% continued the pregnancy and 1% reported illegal abortion (self-report by patient) in the society of this research (Okonofua, Odimegwu, Ajabor, Daru, & Johnson, 1999). In a research conducted in Tehran to determine the prevalence of abortion, it was shown that among %19.5 of those who have had abortions, 95% had unwanted pregnancies. Although 20% of them thought of abortion, just 7.3% actually committed abortion and they claimed that reasons for not committing abortion were fear of mortality and side effects, fear of law, disagreement of the spouse, others’ disagreement, not having facilities for abortion, final side effects and in 52.6% of the cases, the reason was religious beliefs (Okonofua et al., 1999).

Several studies have revealed that the prevalence of depression is 13% after delivery and factors such as depression during pregnancy, anxiety about taking care of the infant, pressures of life, relationship with the spouse, interference with physical disorders, both in the mother and her baby have a role (Nakku, Nakasi, & Mirembe, 2007). Another finding in this study was family and socio-econimoic problems such as husband’s abusive behavior and inability to nurture children. The results indicated that the participants had experienced family issues, and some of them were blamed by their husbands because of not being cautious; as a result, their husband thinks their wife is responsible for unintended pregnancy because they don’t use a safe method of contraception. Studies showed that good relationship with the spouse in women with unwanted pregnancy was low (Akbarzadeh & Khorsandi, 2000). The spouse’s support is an absolutely important issue in maintaining psychological health during pregnancy and it is necessary to attend a special training program by husbands of pregnant women. The husband must be aware of his wife’s emotional needs with unwanted pregnancies, to adapt more to the pregnant women and reduce their anxiety (Kroelinger & Oths, 2000). In Kenya, 65% of men believe that both women and men are responsible for family planning (Were & Karanja, 1994). According to Mistik’s research (2003), 66.7% of men believe that a decision about family planning is a mutual duty of spouses (Mistik, Nacar, Mazicioğlu, & Cetinkaya, 2003). A cross-sectional study in Tehran on the teachers’ knowledge and attitudes of men showed that about 90% believed that decisions about the use of contraceptives should be a common trend between men and women (Tavakoli & Rashidi-Jahan, 2003) so couples need to be trained in decision making and planning (Iklaki, Ekabua, Abasiattai, Bassey, & Itam, 2004). If the required facilities are provided for men, it seems that men will appreciate. Thus, the key puzzle is in the hand of health care providers, not consumers. It’s certainly a need to focus on men like women as an inevitable need, and they will cooperate if they are asked to. Involving men in family planning leads to an increase in their use of contraception, and will encourage more women to use family planning devices (King, Mann, & Boone, 2010; Nasreen et al., 2012). In one study, it was shown that although 70% of married women used modern techniques of contraception, they have not yet found their position among Iranian women (Simbar, 2012)And the young women should be trained about modern techniques to reduce unwanted pregnancies or illegal abortions (Barooti, Sadeghi, Karimi-Zarchi, & Soltani, 2011; Erfani & Yuksel-Kaptanoglu, 2012) So family planning is improved by teaching the use of modern contraceptive devices and in this regard, more specialized education is needed. As reported in studies, the most common abortions in people was spontaneous or accidental. Although intentional-criminal abortion was very close to it, the most common reason of such abortion is the occurrence of unwanted pregnancy (Iranfar, Shakeri, Ranjbar, NazhadJafar, & Razaie, 2005). The most important and common physical disorder resulting from unplanned pregnancy in infants is their weight which is less than 2500 grams and early birth. Other side effects are the probability of congenital anomaly, intrauterine fetal death, early neonatal death, and infants afflicted with diseases like HIV, HBS, HCV and other maternal infections (D’Angelo, Gilbert, Rochat, Santelli, & Herold, 2004; Nasreen, Kabir, Forsell, & Edhborg, 2010). In a research, the results showed that in crowded families with low income abusive behavior is more observed and it seems that economic issues are truly problematic and this matter could be quite effective in sensitizing the couples towards unwanted pregnancy (Hotchkiss, Eckert, & Macintyre, 2000). Actually, after the baby is born, the mother realizes that she has a new responsibility which is taking care of the baby. Accepting such serious responsibility on one hand and limitations in social relationships on the other hand make her prone to disorders (Bergant, Heim, Ulmer, & Illmensee, 1999). In unwanted pregnancies, mothers are exposed to several risks such as lack of care during pregnancy, malnutrition, consuming drugs to abort, stress and mental pressures, etc. (Ramírez-Zetina et al., 2000).

## 5. Conclusions

This study showed that mothers with unwanted pregnancy are under various pressures. In order to prevent unwanted pregnancy and negative experiences, the following points are recommended:

In Iran like other Muslim countries, abortion in limited cases is accepted by the health system. However, some women who have unwanted pregnancies seek illegal abortions, which can lead to unsafe and life-threatening complications. To deal with such a situation, health care providers need to support women through professional consulting. Religious sites (before pregnancy and before abortion) are provided to reduce unwanted pregnancy.

- In unintended pregnancy, both mother and her baby are at risk for physical and mental health. There is a need for medical care and psychological counseling, especially for mothers.

- Most husbands are opposed to abortion in most cases and the psychological pressure is doubled for women with unwanted pregnancies. There is a need to hold specific educational programs for men on how to deal with unwanted pregnancy in mothers.

-A significant percentage of women younger than 18 or older than 35 years are extensively exposed to unwanted pregnancies. The need to increase awareness of premarital education for adolescents and the use of emergency contraception for both are under 18 and over 35 years of age.

Finally, given the emotional and psychological concerns of mother to fetus during unwanted pregnancy and the importance of maternal-fetal and neonatal attachment, prenatal classes are necessary to be held for mothers.

The importance of parents’ involvement in physical and mental health of the fetus and newborn should be emphasized. In addition, the mother – fetus and neonatal attachment promotes physical and mental health in adolescence period.
